# Navigating physical activity after a nerve injury in the arm and hand

**DOI:** 10.1371/journal.pone.0341801

**Published:** 2026-01-30

**Authors:** Linda Evertsson, Cecilia Mellstrand Navarro, Christina Turesson

**Affiliations:** 1 Department of Clinical Science and Education, Karolinska Institutet, Department of Hand Surgery, Södersjukhuset Hospital, Stockholm, Sweden; 2 Department of Clinical Sciences Danderyd Hospital, Karolinska Institutet, Section for Orthopaedics, Stockholm, Sweden; 3 Department of Health, Medicine and Caring Sciences, Division of Prevention, Rehabilitation and Community Medicine, Linkoping University, Norrkoping, Sweden; Neighborhood Physical Therapy, UNITED STATES OF AMERICA

## Abstract

Peripheral nerve injuries in the arm and hand can lead to significant challenges, impacting every aspect of a person’s life. Still, rehabilitation largely focuses on hand exercises, emphasizing motor function recovery. Targeting sensory relearning poses a significant challenge for the brain, demanding neural adaptation and reorganization. While, physical activity is not a standard component of current rehabilitation, yet it supports recovery and promotes nervous system health across other various conditions. However, it remains unclear whether nerve injuries affecting the arm, hand, or fingers contribute to decreased levels of physical activity. Qualitative methods, specifically semi-structured interviews with content analysis, were used to gain a deeper understanding of participants´ experience of change in physical activity after a nerve injury to the arm and hand. Data from in total 20 participants with different levels of nerve injury in the arm and hand were analysed with content analysis. Two themes emerged ‘barriers’ and ‘facilitators’ for being physically active after the nerve injury. The themes displayed three dimensions: internal factors (e.g., prior experience of physical activity and personality traits), physical factors (e.g., pain and hand function) and external factors (e.g., support from family and health care professionals). Key findings indicate that a nerve injury to the arm and hand leads to decrease in physical activity, regardless of injury severity. Previously inactive individuals faced barriers too significant to overcome independently, highlighting the need for targeted support to facilitate physical activity following nerve injuries. These findings may offer new insights into potential rehabilitation strategies.

## Introduction

Patients with a nerve injury in the arm and hand encounter a spectrum of challenges that extends across physical, psychological and social dimensions. Contemporary rehabilitation following nerve injury predominantly focuses on hand-specific exercises, targeting motor recovery. The integration of sensory relearning strategies introduces additional challenges for the brain. These techniques demand early neural adaptation and reorganization, particularly during the acute phase of injury. A reduction in physical activity (PA) may hinder and negatively impact this rehabilitation process. PA refers to any bodily movement that requires energy expenditure [1]. It incorporates various forms of movement, including exercise, sports, active recreation, work-related activities, household chores, and any other daily activity that involves physical effort [2,3]. A broader definition of PA encompasses people moving, acting, and performing within culturally specific spaces and contexts, influenced by various interests, emotions, ideas, and relationships [4].

Levels of PA can change for various reasons due to physical injuries, illness, disability, life events and psychological reasons [5]. Patients with a nerve injury often experience pain, depression and anxiety after the injury [6,7], which are all factors potentially reducing the level of PA [5].

Physical exercise offers multifaceted benefits to the nervous system [8] and has been identified as an effective therapeutic approach for various physical diseases and metabolic dysfunctions [9,10] and psychiatric conditions [11,12]. PA has been shown to improve cognitive functions and expand learning opportunities [13]. Available rehabilitation regimens after a nerve injury are limited [14]. Most often rehabilitation is restricted to hand exercises or sensory relearning techniques. They involve neoplastic abilities of the brain and aim at improving hand function and its use in daily activities [15]. PA is not a part of traditional nerve injury rehabilitation.

To what extent PA levels alter after nerve injury is still largely unexplored. Therefore, this study aims to investigate potential changes in patients’ experiences of PA after a nerve injury in the arm and hand.

## Method

### Study design

This is a qualitative interview study that explores the experience of changes in physical activity (PA) after a nerve injury in the arm and hand. Data was analysed using content analysis [16,17].

### Research context and researchers’ position

This study was performed at two specialized hand surgery clinics in Sweden, both offering in-house rehabilitation for nerve injuries. Rehabilitation typically extends over long periods, sometimes lasting several years, to support functional recovery, however varies based on location and individual needs.

The interviews were conducted by a physiotherapist and an occupational therapist, each with over 10 years of experience treating patients with nerve injuries. To avoid bias, none of the interviewers had previously treated the study participants.

### Participants

Patients were recruited using a relevance sampling strategy [18,19] also known as purposive sampling [18] and identified in medical journals at Stockholm or Linköping hand surgery clinics. Eligible participants were individuals aged 18–75 years at injury, treated for a nerve injury in the upper extremity one to three years prior to the present investigation.

Participants with varying ages, sexes, and nerve injury locations (including injuries affecting the fingers, hand, forearm and shoulder region), were included to provide a broad spectrum of experiences, enriching the dataset. Additionally, individuals with varying prior experiences of PA were selected to ensure a diverse range of perspectives and experiences. Exclusion criteria were difficulties in communication in Swedish or English making an interview impossible.

Eligible participants were approached during outpatient visits or contacted by telephone. Twelve eligible participants could not be reached by mail or phone.

Oral and written information regarding the study was provided by the treating therapist and written consent was obtained from each participant before the interview was conducted. Participants ranged in age from 23 to 75 years, with a median age of 49 years (SD 17,81). All participants, but four with plexus injuries, underwent surgery in direct connection with their nerve injury; no participants underwent tendon transfers before interviews. Background data of the 20 participants are presented in Table 1.

**Table 1:** Study participants (n = 20), injured nerve, injury site and participants not treated surgically at the time of injury.

Participant	Sex	Dominant side/ Injured side	Injured nerve*	Interview in minutes	Interviewer
P1	F	Right/right	Median/ulnar	67	LE
P2	M	Right/right	Median	42	JS
P3	F	Right/left	Digital	39	LE
P4	M	Right/left	Plexus ^π^	38	LE
P5	F	Right/right	Median/ulnar	52	LE
P6	F	Right/right	Median	53	JS
P7	F	Right/right	Plexus ^π^	49	LE
P8	M	Right/right	Plexus ^π^	34	LE
P9	M	Right/left	Digital	33	LE
P10	F	Right/left	Plexus ^π^	40	LE
P11	M	Right/left	Digital	27	LE
P12	M	Right/right	High median	36	LE
P13	M	Right/right	Ulnar	64	LE
P14	M	Right/left	Digital	43	LE
P15	M	Right/left	Median/ulnar	72	LE
P16	M	Right/right	Plexus	43	LE
P17	F	Right/left	Median/ulnar	29	JS
P18	M	Right/right	Plexus	42	JS
P19	F	Right/left	Digital	28	LE
P20	M	Right/right	High median	39	LE

*Digital: volar nerve injury in the palm or finger. Median: nerve stem injury at wrist or forearm level. Ulnar: nerve stem injury at wrist or forearm level. High median: nerve stem injury at elbow level. Plexus: brachial plexus injury at shoulder level including all three branches (median, ulnar and radial nerve). ^π^ Not treated with surgery immediately after injury.

### Data collection

A semi-structured interview guide was developed with questions about PA; level before and after the nerve injury and the possibility to be physically active today (1–3 years after the nerve injury). We conducted a pilot test of the interview guide with two persons and these interviews were also included in the study. Based on these pilot interviews, we made slight revisions of the interview guide to improve question phrasing and flow. The finalized interview guide is included as an appendix in the manuscript for full transparency. The interview guide was used to direct the interview towards different areas in life.

The interviews were conducted during the period 16 April 2021–8 January 2025 by the first author (LE), occupational therapist, or a physiotherapist and researcher colleague (JS) familiar with the patient group, study aim, and interview technique. Face to face interviews were conducted with four participants. All other participants were interviewed over the telephone due to location of living or Covid restrictions during the study period. The interviews lasted from 27 up to 72 minutes with a mean length of 44 minutes. Data collection ended after 20 interviews, as no new information emerged during the final 4–5 interviews. This indicated that data saturation had been reached, supporting the adequacy of the sample size for capturing the range of perspectives relevant to the study.The audio-recorded data was transcribed verbatim by LE, a medical secretary or an occupational therapist colleague not involved in other aspects of the study. The participants’ names and specific details in quotes were removed to protect the participants’ identities.

### Data analysis

Initially, the audio-recorded data from each interview were transcribed verbatim and the interviews were read several times to enable the researchers to familiarize with the data. Data was analysed with qualitative content analysis [16,17] with the emphasize on experiences of changes in PA after nerve injury in the upper extremity. As a first step, each interview was analysed separately (LE, CMN, CT). The process started with identification of meaning units related to PA and the change in PA as expressed by the participants. The meaning units were condensed, interpreted, and labelled with codes close to the text trying to capture the essence [17]. Groups of codes were compared among the research group participants, and the interrelationship identified was used to create themes that encapsulated the commonalities found.

When analysing the codes, recurring trends from the participants’ wordings emerged, and these were either positive or negative features of experiences for being or not being physically active. The two distinct sides, whether to be involved in PA or not were identified and labelled as barriers and facilitators. Further analysis of the codes and content of barriers and facilitators resulted in the identification of subthemes. The interrelationship of the subthemes under each theme, were reviewed and discussed among the researchers until agreement was reached [16,20].

The analysis was an iterative work process, done in sequences of individual work by LE and followed by discussions in the multi-disciplinary research group, two occupational therapists and one surgeon with experience of qualitative research (LE, CT, CMN), to refine the analysis and ensure that the findings were grounded in data.

### Ethical considerations

This study was conducted according to the ethical principles outlined in the Declaration of Helsinki (World Medical Association, 2008) and was granted ethical permit by the Swedish Ethical Review Authority, Dnr 2020−05884, Dnr 2021−04528, Dnr 2024-01913-02. The study was registered at clinicaltrials.gov, ID: NCT05080608.

## Findings

The results demonstrate that all participants experienced changes in PA, with decreases or increases occurring over different time spans and to varying degrees. This finding was evident among all participants despite large discrepancies in the severity and location of their nerve injury. Reduced PA immediately following the injury was often evident and communicated as a complete disruption of the daily routine.

P5 *“When the injury happened my whole life was just put on pause, uh, my private life, my training definitely, my work life. It was like … within the space of a nanosecond…” (Median & ulnar)*

**Fig 1:**
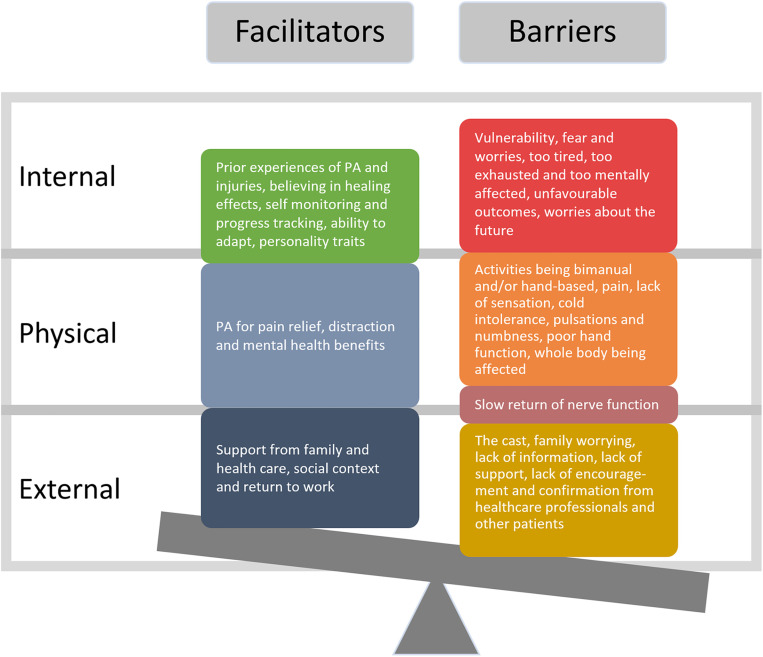
Barriers and facilitators to physical activity: Themes and subthemes across internal, physical, and external dimensions. The two themes, barriers and facilitators for being physically active after a nerve injury, and their subthemes illustrated with the three dimensions: internal, physical, and external factors.

Two recurring themes emerged and were repeatedly mentioned in the interviews: ‘barriers’ and ‘facilitators’ for being physically active after the nerve injury. The themes barriers and facilitators for being physically active comprised several subthemes which exhibited similar characteristics, and these were identified as three dimensions: internal factors, physical factors, and external factors (Fig 1). The themes and subthemes are presented based on these dimensions.

### Internal barriers and facilitators

The internal dimension of barriers and facilitators reflects participants’ challenges and inner strengths, with barriers often described as **vulnerability, fear and worries**. The days or weeks immediately following the injury were by most participants proposed as the utmost inactive and vulnerable stage. In the initial stage participants described to be lying in the sofa, often in front of the television the whole day. Fear was communicated to contribute to their sense of vulnerability, with concerns and worries about PA potentially triggering pain, exacerbating existing symptoms, or causing further harm to the injured limb.

P6 *“I also had — I know — a fear of using my hand, like somehow, because it was a trauma, I was afraid that something might break” (Median)*

This was similarly communicated after digital nerve injuries;

P3 *“as if I touch something with the tips of my fingers, but feel it all the way … like a jolt all the way down to the palm of my hand. Because I’m so afraid of bumping my hand into something” (Digital)*

In several cases participants became more aware of any physical danger after the injury. This fear even made participants hesitant to leave home or use public transport, limiting their ability to engage in previous activities or try new forms of PA. This sense of vulnerability led some individuals to use additional protective gear and splints. Along with this vulnerability, participants commonly described to be **too tired, too exhausted, and too mentally affected** to even consider to be physical active. When reflecting on their inactivity, participants consistently emphasized the nerve injury’s impact on the brain. **The mental effects** contributed to internal barriers hindering activity. One participant vividly described this shift as:

P18 *”No, but it’s more complicated — I’m definitely more tired now, and my brain has clearly been affected by all this, so I would say that fatigue is a major reason why my physical activity has decreased” (Plexus)*

Additionally, participants reported that the injury led to negative psychological effects, reinforcing mental barriers to PA. Moreover, when participants labelled the injury as a very traumatic event this was contributing as an internal barrier. Some participants expressed that their life flashed before their eyes while injured. Such dramatic experiences were clearly mentioned especially after major nerve injuries, plexus injuries and concomitant major bleeding at the site of injury. In contrast, some described the initial postoperative period in a cast as a break or “vacation,” enjoying the lack of expectations to be active and feeling no urge to resume PA. Descriptions from participants with digital nerve injuries varied, and informants rarely saw the injury as life-threatening, though emphasized the mental and physical challenges of staying active.

P19 *“I think it was the shock — one second I had all my fingers intact, and the next second, it was like, I had a lifelong injury […] I mean, the hardest part — I think maybe it took about six months before I could properly start gripping with weight-bearing. It just didn’t feel right, you know” (Digital)*

Exhaustion, fatigue, passivity, and vulnerability lasted from days to years, with some still affected during the interview. In the acute phase, participants sought distraction from distressing thoughts and fear of pain. Additionally, reduced enjoyment led participants to abandon activities when they were unable to perform PA as before.

P12 *“I’ve felt defeated and have given up both playing padel and golf. I’m not the kind of person who gives up, but I’m not going to keep doing something that doesn’t feel fun anymore, you know” (High median)*

Similar expressions of lack of motivation were noted among participants with digital nerve injuries;

P14 *“the injury became, like, a kind of a break [from PA], and then I didn’t really feel like I wanted to go back to it”* (*Digital)*

Active participants, on the other hand, credited awareness from **prior PA experience**, and past injuries as key factors. These experiences were articulated as beneficial internal facilitators while adapting to prior activities;

P14 *“when I came back from the hand injury I did a climbing training course that was very easy on your hands and I feel that I’ve become a lot stronger after that course, even though it was low intensity”* (*Digital)*

As noted by formerly active participants, this could even facilitate engagement in new activities. Despite the grief of lost functions, prior PA experience was credited with facilitating an earlier return to activities. Despite prior experience with PA, participants reported abandoning their previous activities when faced with **unfavourable outcomes**. Meanwhile, others with pre-injury PA experience cited **self-monitoring and progress tracking** as internal facilitators. Observing the return of functions through self-monitoring was described as an internal motivator for maintaining activities. Prior exercise habits also facilitated activity adjustments, motivating participants to stay more active. Whereas prior inactive participants expressed doubt about returning to activities, pre-injury exercisers expressed greater belief in their ability.

P14 *“I feel that I’m probably also more, like, you become more resilient just because you stay active, and that maybe it’s easier to bounce back” (Digital)*

Several participants identified PA as promoting **healing effects**. When noticed, this too facilitated motivation for an earlier return to activities. Reasoned by one participant as:

P13 *“I think the basic principles or the benefit that physical activity gives to mental health are as true if not especially true in this context. I think... Just sitting at home and waiting for it to heal … does not really do you any favors because this process is so long” (Ulnar)*

In line with its healing effects, participants emphasized the potential of PA as a key component of rehabilitation after arm and hand nerve injuries.

**Personality traits** such as being stubborn or having a positive view, patience and constantly trying in life was discussed as valuable internal facilitators to stay active. Some of the traits were discussed to emerge from upbringing, nurture, and innate characteristics, all of which were identified as influential factors in determining whether someone is active.

P15 *“my mother was really driven so that’s probably where I’ve gotten it from, the fighting spirit … it’s probably partly from mom … this sort of driven mind-set to never give up and to keep fighting” (Median & ulnar)*

Active participants also expressed appreciation for their prior PA and the mind-set that comes with activating the body and challenging the brain physically, recognizing the internal mental facilitator for being active. Regardless of PA experiences feelings of **worries about the future**, and the new arm and hand abilities were internal obstacles. A lack of hope and increased anxiety were described as factors undermining the motivation to resume activities.

P5 *“I was really anxious because I was afraid about what my future would look like after the injury I went through” (Median & ulnar)*

### Physical barriers and facilitators

The physical dimension of barriers and facilitators reflects the physical elements, and symptoms which influence the ability to stay active. All participants mentioned physical barriers as reasons for reduced PA. Various physical barriers for not being able to stay active after the injury were expressed in relation to the arm and hand and to **activities being bimanual**. Additionally*,* nerve healing was often expressed as an unchangeable physical barrier, with participants emphasizing that adapting to their new arm and hand would take time and delay their return to PA. In unison inability to perform **hand-based activities** was articulated. Reasoned by one participant as;

P13 *“yeah I mean so many of my activities were hand based, you know so it was like cycling and backpacking are my two main things. And backpacking I think I can, I can do it now fine, and I could probably start try to cycle again I think I could start… “(Ulnar)*

Symptoms were experienced even to the extent that participants stopped attempting previous activities due to reduced hand function. Frequently the concentration required to focus on hand function after the injury, was mentioned to affect physical ability.

P18 *“it’s mostly that I have to concentrate on which damn muscles I’m trying to activate more than the physical effort itself” (Plexus)*

Swimming, skiing, horseback riding were examples of activities participants mentioned giving up, due to not being able to use the hand and arm. One obstacle when swimming expressed from a participant with a plexus injury was the whole function of the arm. While for a person with a digital nerve injury a reason would more often be explained as the temperature of the water causing pain. This shift in not being able to use the hands in PA was further described to lead to poor fitness after the injury;

P10 *“Before we used to go for a swim. It’s also things like this that are totally impossible today. But what we do now is that we go out for a walk. And that doesn’t really give you the same thing… but of course it gives you something. But it’s just not at all the same kind of thing as I did before and not as often because, I get really tired and then some days I have really bad pain in my hand” (Plexus)*

In unison participants with **pain** expressed this as a major physical barrier for PA, and the reason for having a more sedentary lifestyle. Pain as a physical barrier was described to not only affect the hand and arm but the whole body after the injury.

P16 *“Well, you were in pain and your arm was paralyzed, and at first, you didn’t even want to move the rest of your body, because that also felt uncomfortable” (Plexus)*

Participants with nerve injuries in the arm and wrist level often described pain affecting all aspects of life, while after finger nerve injury pain was often linked to barriers of lack of grip function, sensation and specific activities. **Lack of sensation** and cold intolerance in the arm and hand were physical barriers universally mentioned to contribute to discomfort, avoiding PA or even resulting in staying indoors during cold weather. When a nerve in the finger was injured, participants particularly described avoiding using that finger, while with an injury to the plexus, the interpretation of temperature resulted in a physical barrier, causing a complete shift in PA. **Pulsations and numbness** were reported from participants with varying levels of nerve injury as adverse symptoms, which could escalate when being physical active.

P20 *“No, I tried running one time … in the late autumn, then I ran quite a long way … a little over 10km but then […], haha, I have basic fitness level from boxing, but then I noticed that my whole arm, from the injury down to my hand had become numb” (Median)*

Equally noticed by participants with a nerve injury more distally in the fingers;

P11 *“I [thought I] should try to go play table tennis but it didn’t work. I played a little with my left hand and stuff, but it didn’t work because when I moved a lot, my hand started to pulsate” (Digital)*

On the contrary others experienced the opposite with reduced pulsations and numbness when being physically active for a longer period of time:

P2 *”It lasts for maybe an hour or two afterward, when I have a calm moment where my hand feels okay — like, I don’t have that constant pulsation, and the numbness hasn’t been there, you know” (Ulnar)*

Participants who experienced symptom relief in the hand found it easier to stay active. Several mentioned that engaging in PA not only reduced hand symptoms but also stress, furthermore, helping manage negative thoughts and alleviate pain perception. Participants found it easier to resume activities not involving the hands like walking. However, several faced walking barriers, often attributing them to the whole body being affected from the injury.

P17 *“I should have stuck to more strength training but I have, I’ve just been afraid of over-exerting my body in some way, I’ve been, like I said, pretty worn out physically and mentally tired and that has just prevented me from getting going” (Median & ulnar)*

However, water exercises were expressed to provide hope when other activities failed, particularly aiding in the gradual recovery of arm function after brachial plexus injuries. Others questioned their PA level after the injury and questioned their sedentary lifestyle during the interview. When reflecting on their lack of PA after the injury, participants struggled to fully understand how they had ended up leading such an inactive lifestyle.

### External barriers and facilitators

The external dimension of barriers and facilitators uncovers influences from the environment that affects one’s ability to stay active. Several of the obstacles that led to inactivity, or made activity impossible, were described as being outside the participants’ control. **The cast** was consistently mentioned as an example of an external barrier which limited PA. A wide variation in information about restrictions during the cast period was noticed among the participants. Some described being encouraged to go for walks while others only received restrictions in relation to the injury; i.e., *“you can’t/ you are not allowed to…”.* In addition, an external barrier expressed could be the surrounding and family worrying. This, along with the shift from independence to reliance on family and healthcare professionals, was described.

P1 *“One’s everyday life changes; you become dependent on others. And even if you can still manage certain things when you end up there, as it deepens more and more, it becomes very difficult to bring yourself back to the surface again*” *(Median & ulnar)*

Many participants attributed their sense of lost control after the injury to a **lack of information.** Frequently articulated as *P1 ” Don’t know what I’m allowed to expose myself to”*. Ambiguous information from health care professionals was described to raise feelings of insecurity. During the interviews, participants sought advice on rehabilitation and restrictions, highlighting their need for knowledge. The lack of informed guidance led participants to be overly cautious, often protecting and restricting the use of their arm. This shift to a more sedentary lifestyle due to insufficient knowledge was evident.

P13 *“… I do not do anything I just game and stuff like that. Prior to this I was not doing this at all I was super active I was not doing it at all but I definitely picked it up in the aftermath because I felt like I had nothing else to do I did not know what to do with myself…” (Ulnar)*

Furthermore, participants articulated that a lack of knowledge was linked to lower energy, with increased pain, and reduced activity, including gym attendance and running, due to unclear restrictions. Most participants described a **lack of support, encouragement and confirmation** in being active **from the health professionals**. Some participants, encouraged by health professionals to go for walks saw it as vital to resuming PA. Some noted progress on their own and could adapt their PA. Those advised to “be careful” described to be more cautious, while others resumed activity once their cast was removed. External support from healthcare professionals, family, and friends was expressed to facilitate activity.

P6 *“My husband has been a huge support in all of this — both he and my son, you know, encouraging me, saying things like ‘Come on now, we’ll sort this out,’ and ‘Nothing is hopeless,’ and so on. And then the rehabilitation, when I got to go to the hospital and meet the physiotherapist, the occupational therapist, and the counsellor, and was able to talk things through and see the possibilities […] So I think it’s a combination of a lot of support from home and also strong backing from the hand surgery unit” (Median)*

External support also boosted the courage to resume previous activities despite fear, as one participant stated:

P17 *“Partly, I had people around me who encouraged me, and then I also gathered my courage. But I had also understood the benefits before I got back up on the horse again and what it could give me, so really it was just about overcoming the fear” (Median & ulnar)*

Various **social contexts** emerged from participants as factors influencing activity levels, such as participating in sports clubs or gyms. Participants with pets noted them as external facilitators for going outside and staying active. A shift in context occurred when a participant, in a new cultural context among war-experienced individuals with severe injuries, was encouraged to go to the gym for the first time after injury. The reflections in mind-set were:

P20 *“here in Sweden people react in an entirely different way to this type of injury than how they react in countries that are familiar with war” (High median)*

In line with social contexts, participants asked to **meet other patients** in the same situation for support to resume PA.

P6 *“… to get a reminder that your legs are working … this is difficult because of confidentiality and stuff … but this thing with having some kind of role model” (Median)*

Work context was frequently discussed in relation to PA, serving as both a barrier and a facilitator. To be given more time when on sick leave was sometimes a new context mentioned as a beneficial external factor to stay active. The extra time was described as positive and necessary, as hand-based activities took longer and required adjustments. Other external facilitators included **return to work** along with schedule adaptations for an early return, noted by one participant:

P18 *“I’m not only, I’m not just a great lump on the sofa anymore but now I can actually return to some work, take on some tasks that I had before the accident … so that’s what motivates me to go to work and set my alarm for 05:45”* *(Plexus)*

## Discussion

This qualitative study explores the impact of nerve injuries in the arm and hand on physical activity (PA), identifying barriers and facilitators influencing participation in PA during recovery. None of the participants maintained the same level of PA following their injury. The main findings revealed that participants shared similar perspectives on changes in PA, with reduction of activity level even when the injury involved the digital nerves, which primarily are sensory nerves that serves a restricted area. Most participants with more proximal level of nerve injury in the hand and arm reported a more life-altering impact.

Our study highlights the early post-injury stage as the most critical and sedentary period after a nerve injury in the arm and hand. This is similarly reported after lower extremity injuries [21], emphasizing the importance of identifying inactive patients at an early stage after injury. Several diseases show improvement using PA as an intervention [9,22], along with cognitive benefits of exercise [23]. Our participants noticed reduced motivation and emphasized the injury’s impact on the brain as a barrier to staying active, indicating that the nerve injury might not only impact PA levels, but also complicate early relearning regimens involving the brain’s plasticity mechanisms directly after the injury. Moreover, these findings underlines the need for support to achieve the WHO guidelines on PA [2] early after injury. We suggest screening for both depression [7], and inactivity, as both appear to be risk factors after upper extremity nerve injury. To our knowledge, this is the first article exploring PA after nerve injuries in multiple levels of the arm and hand. Incorporating PA in the rehabilitation regimen after these injuries could improve quality of life, nerve rehabilitation and potentially enhance nerve regeneration.

Several reasons for inactivity have been identified [24]. Both pain and psychological distress have been reported after nerve injuries in the arm and hand [25]. This underscores the importance of support, as participants in our study found pain to be a considerable barrier, compounded by internal worries, described as difficult to overcome alone. Further, pain is reported to contribute to a fear of movement (kinesiophobia) [26]. This is in line with our study that identified several barriers related to kinesiophobia, such as vulnerability, fear of re-injuring or exacerbating their injuries by being active. A unique aspect of our study is the inclusion of participants with digital nerve injuries, which adds a novel perspective to the understanding of recovery and PA engagement. Pain and feelings of fatigue also impacted our participants mental well-being, contributing to a lack of motivation preventing engagement in PA. However, individuals with more PA experience adapted and resumed activities more easily after injury. Reports indicating more active individuals less likely to develop depression [11], In lights of this, considering prior activity level in rehabilitation may help reduce psychological stress and identify those needing support to stay active after injury [27]. However, the beneficial effects of PA after peripheral nerve injuries in the upper extremity are still relatively unexplored in humans. Regular PA is strongly linked to better brain health and cognitive function in humans [28]. PA serves as a powerful therapeutic strategy in managing non-communicable diseases, promoting recovery and enhancing overall health outcomes [29,30]. While most investigations on PA have been conducted in rodent studies indicating beneficial effects on nerve regeneration [27], human studies show that aerobic training increasing biological markers for nerve regeneration [28,31–34]. Hence, the barriers and facilitators identified in our study could inform the development of new interventions after nerve injury in the arm and hand including PA.

Another change in activity noticed in our study was an increase in TV watching after the injury. One study found evidence linking screen time to depression, with a risk of low self-esteem [35]. Depression [7] and post-traumatic stress (PTSD) [36] have been identified after nerve injuries. Linking PA benefits to mental health, our study suggests supporting inactive patients’ post-injury may reduce the psychological burden. The Self-Determination Theory (SDT) highlights autonomy, competence, and relatedness in motivating PA [37]. These findings align with emerging evidence that PA and exercise interventions not only support physical rehabilitation but also play a critical role in improving mental health and resilience [38], as demonstrated in studies which highlighted the integration of PA in mitigating depression and suicide risk through neurobiological and psychological mechanisms [39]. By fostering intrinsic motivation in inactive patients through patient-centred approaches, healthcare professionals can promote PA, improving overall health and reducing healthcare costs.

Cold intolerance is linked to pain and frequently reported after nerve injuries [40,41]. Our study indicated activity restrictions due to temperature sensitivity, both from outdoor exposure and gripping various materials. Persistent pain from abnormal temperature responses was noted year-round, often during outdoor activities, contributing to a change in activities and seasonal variations in PA. Adverse weather conditions, along with factors like age, health, socioeconomic status, geography, and disability, have been shown to limit PA [42]. Yet, limited strategies exist for treating cold intolerance [43] or how to compensate hand function. To facilitate greater PA, further research is needed to improve heating devices and adapt activities to minimize their impact on grip and hand function.

While our study aligns with previous research on disability, it highlights internal barriers. Key internal barriers to physical activity (PA) included fear, low energy, and lack of motivation. Conflicting information about restrictions during nerve healing added to these challenges, with unclear advice increasing fear, pain, and feelings of losing control, ultimately leading to reduced PA. This aligns with the fear-avoidance model [44] suggesting responses to pain can vary depending on psychological interpretation of symptoms. According to the model some patients see PA as threatening and avoid it, which can worsen symptoms, while others view PA beneficial and experience pain relief. These findings were also observed among our participants, whereas some found PA beneficial in reducing symptoms, others did not. These individual variations may be due to differences in pain perception, past experiences with PA, or differences in our nervous system. The findings of Goubran et al. (2025) underscore the negative association between kinesiophobia and PA, highlighting the importance of integrating psychological support into rehabilitation programs to address fear of movement and encourage patients to remain active [45]. While exercise might improve handgrip and strength [46], still, the relation between exercise and our hands are relatively unknown. Thoughtfulness needs to be put into rehabilitation after nerve injuries in the arm and hand. Despite recognizing its benefits, most participants did not engage in regular PA after injury or meet recommended guidelines [2].Our findings highlight the need for healthcare professionals to provide psychological support and to promote tailored safe PA during recovery after nerve injury in the hand and arm.

The context in which PA occurs can significantly impact our activities [4]. Our study highlights various barriers and facilitators, reflecting how different environments influence PA participation. Cultural and societal views on injuries, along with past traumas and war experiences, was reported to influence the PA attitudes. Whilst families have similar roles across cultures, non-Western cultures tend to place greater emphasis on family. Social and contextual factors have previously reported to influence PA [47] and a companion can boost PA levels [48]. Our participants called for support from health care professionals and to meet others with the same injury to learn from their experiences to be more active after the nerve injury.

### Strengths and limitations

The present study has several strengths and limitations. One limitation is that inclusion may have been biased toward participants with a greater predisposition for engaging in PA. However, no one invited to an interview declined to participate. Including participants with different levels of PA experience and varying nerve injury severities broadened the range of perspectives and strengthened the data. However, we acknowledge that this also might be a limitation. While this approach increased clinical relevance, the heterogeneity of injury types may have limited the specificity of our findings for distinct patient subgroups. Patients with similar nerve location injuries can also have vastly different treatment options and at varying time points after the injury which may affect patient experience through their recovery and perceptions of barriers/facilitators. Despite potential variability, clear similarities were found across different injury levels. Future research focusing on more homogeneous populations, such as professional athletes or patients with isolated hand or finger injuries, would provide valuable complementary insights. The multidisciplinary research team provided valuable perspectives, enhancing the study’s trustworthiness. A key strength is the authors’ diverse professional backgrounds and knowledge of the patient group and PA, which enriched the interpretation of the results. For example, discussions were held around how clinical expectations of recovery might differ from patients’ experiences, and how terminology used by participants could be interpreted differently depending on professional background. However, awareness of the preunderstanding of the researchers performing data collection and analysis was also recognised and discussed to ensure findings were grounded in data. To strengthen the credibility of the results, the authors discussed the interpretation of data, categories and subcategories throughout the analysis process, to reach a consensus and ensure they accurately represent the data [49]. Another limitation was that participants were interviewed at different time-points after the injury, which may result in recall bias. As the study is based on participants’ retrospective accounts, their descriptions may be influenced by memory limitations or changes in how they interpret their experiences over time. This type of recall-related bias is important to acknowledge, although such subjective reflections are also central to qualitative research. However, the passage of time may have allowed participants to reflect on their injury and recovery with greater emotional distance, potentially leading to more positive or negative reinterpretations than they might have expressed closer to the time of injury. It is also important to note that the events discussed were often significant and impactful, which tends to enhance memory retention and the depth of reflection. The variation in time since injury therefore contributes to capturing a broader spectrum of experiences, which enriches the data. Other limitations are the lack of information about the participants pre-injury PA level or available social and environmental support. Further, as most participants were over 30 years of age, the findings may primarily reflect the experiences of older patients and may not fully apply to younger individuals with upper limb nerve injuries.

Initially, the COVID-19 pandemic’s impact on the interviews was observed, redirecting discussions towards its effects on PA. This led to a temporary postponement of data collection, as the pandemic’s influence on responses potentially diverted attention from the study’s primary objectives.

## Conclusion

The study reveals a reduced level of physical activity following a nerve injury to the arm and hand. It identifies previously inactive individuals as particularly vulnerable to adopting a sedentary lifestyle. Some participants encountered long-term barriers that were too significant to overcome without external support to resume activity. The main findings regarding physical activity were consistent across participants, regardless of injury severity; however, those with more severe injuries described a more profound and life-changing impact.

## Supporting information

S1 File (S1 Text, Interview guide). (PDF)
