# Supplementary material for: Navigating physical activity after a nerve injury in the arm and hand
Source: PLoS One. 2026 Jan 30;21(1):e0341801. doi: 10.1371/journal.pone.0341801 (PMC12857933; doi:10.1371/journal.pone.0341801)
Supplement: S1 File — (PDF) [file pone.0341801.s001.pdf]

APPENDIX

Interview Guide

Presentation and a short introduction to the definition of physical activity: We will talk about physical activity. This can include all areas of life: daily routines, work, leisure time, and exercise (including structured exercise, everyday movement, and sedentary behaviour), and there are no right or wrong answers.

Introductory Questions:

Patterns of physical Activity– (Before, what, when & how)

- Would you like to tell me what a typical day in your life looked like before the injury?

Patterns of physical Activity – (Today, what, when & how)

- Can you describe when and where you are typically physically active in your daily life today? Please describe whether your activities are (indoor or outdoor, work/home, seasons, days of the week, or times of day)
- Could you describe any differences in the physical activities you engage in now compared to the past?
- What does being physically active mean to you?
- Is physical activity connected to any particular interests, such as individual activities, group sports, or hobbies like gardening? (How important is physical activity to you)

(Here, try to explore differences between before and after the injury)

- How does your family situation, work environment, and the physical demands of your job (if applicable) influence physical activities you engage in?
- Looking back, how do you feel your physical activity has changed since your nerve injury and surgery? Can you describe any specific differences in the types of activities you engage in or how you approach physical activity now?
- If so, in what ways has your nerve injury affected your ability to be physically active and can you describe whether you've changed the way you engage in physical activity?

Closing question:

- Is there anything you would like to add that you feel is important?
